# The Perspective on Secondary Research Practices: A Cross-Sectional Analysis

**DOI:** 10.3390/healthcare13080927

**Published:** 2025-04-17

**Authors:** Piotr Ratajczak, Katarzyna Oziewicz, Isolde Sommer, Dorota Kopciuch, Anna Paczkowska, Tomasz Zaprutko, Krzysztof Kus

**Affiliations:** 1 Department of Pharmacoeconomics and Social Pharmacy, Poznan University of Medical Sciences, 60-806 Poznan, Polanddkoligat@ump.edu.pl (D.K.); aniapaczkowska@ump.edu.pl (A.P.); tomekzaprutko@ump.edu.pl (T.Z.); kkus@ump.edu.pl (K.K.); 2 Cochrane Austria, Department for Evidence-Based Medicine and Evaluation, Danube University Krems, 3500 Krems, Austria; isolde.sommer@donau-uni.ac.at

**Keywords:** secondary studies, systematic review, PRISMA, Cochrane, COVID-19

## Abstract

**Background:** The surge in scientific publications during the COVID-19 pandemic has heightened the need for reliable secondary studies such as Systematic Reviews, synthesising evidence to guide clinical and public health decisions. This study aimed to analyse the current practices, preferences, and challenges faced by researchers conducting secondary studies and assess the impact of the COVID-19 pandemic on these practices. **Methods:** An online survey was conducted among researchers actively involved in secondary research. Email addresses were collected from PubMed for publications related to COVID-19 secondary studies between 2020 and 2022. The survey comprised 24 questions, including single- and multiple-choice formats, covering general information, Systematic Review processes, and changes during the pandemic. Statistical analysis, including Pearson’s Chi^2^ test, was performed on key responses to identify significant correlations. **Results:** This study highlights that only 26.9% of respondents use keyword-generation tools. However, those using PubMed were more likely to utilise MeSH (*p* = 0.01486, df = 1, Chi^2^ = 5.932568). Systematic Review software adoption was prevalent, particularly for Rapid Reviews, with Covidence being commonly used (*p* = 0.00843, df = 1, Chi^2^ = 6.938953), especially during the screening stage (*p* = 0.02400, df = 1, Chi^2^ = 5.094851). Despite this, many researchers still reported that they did not use any software. A total of 94.9% of respondents reported adherence to PRISMA guidelines, and protocol registration was strongly associated with following these guidelines (*p* = 0.00320, df = 2, Chi^2^ = 11.48858). Researchers using Embase were significantly more likely to incorporate RCTs (*p* = 0.00360, df = 1, Chi^2^ = 8.476092), while Cochrane reviewers showed a lower reliance on non-randomised trials (*p* = 0.02601, df = 1, Chi^2^ = 4.955580). During the COVID-19 pandemic, 64.3% of respondents observed a significant increase in secondary studies. **Conclusions:** This study highlights key trends in secondary research, emphasising adherence to established guidelines and the growing reliance on software tools. However, gaps remain in protocol registration and keyword generation practices. Addressing these gaps through targeted training may improve the quality of future secondary studies, particularly during global health crises.

## 1. Introduction

Secondary studies play a significant role in planning treatments and medical procedures and predicting the outcomes of applied therapies. Through comprehensive data analysis from multiple primary or secondary source publications, it is possible to determine if a particular treatment produces specific effects, the frequency of adverse effects, their nature, and their severity. It is also possible to verify patient groups, such as those with a particular disease, on whom a specific drug works best. Through collective synthesis and analysis of data from various sources (not just scientific publications), healthcare decision-makers have access to information to make decisions that are most beneficial for patients and healthcare systems, considering medical and economic perspectives. Examples of such institutions include the World Health Organization, the European Medicines Agency, and the European Network for Health Technology Assessment. Secondary research, based on the principles of evidence-based medicine [1], offers practically unlimited opportunities for analysis, synthesis, and the summarisation of results, thereby providing the foundation for rational decision-making.

The first clinical Systematic Review was published in 1955 in the scientific journal “Journal of the American Medical Association (JAMA)” [2] in the United Kingdom, and since that time, interest in Systematic Reviews has significantly increased. It is estimated that an average of 11 new Systematic Reviews are published daily [3]. In light of the large number of new studies, and aiming to standardise the conduct of Systematic Reviews and disseminate such research, the Cochrane Collaboration was established in the early 1990s. Today, it is one of the most significant scientific organisations creating guidelines, methodologies, and tools for conducting this process [4]. Tracing protocols, decision-making processes, and conducted interventions are crucial to deem a study valuable. It allows for insight into how the study progressed at each stage, thereby verifying the quality, credibility, and reliability of the presented results. Such an approach is only possible when employing specific methodologies based on guidelines such as those provided by the Cochrane Collaboration [5] or other frameworks like The Preferred Reporting Items for Systematic Reviews and Meta-Analyses (PRISMA) [6]. Due to the diversity of research topics and often limited time for analysis, many secondary studies are available, including Rapid Review, Scoping Review, Systematic Review, Umbrella Review, Evidence Map, or Qualitative Systematic Review [5]. The choice of appropriate databases from which we retrieve studies (e.g., PubMed and Embase) also remains significant. Regardless of the specific method chosen, the success of conducting secondary studies depends primarily on a systematic methodological approach and skilful adaptation of particular tools (including computer software) to perform this process.

One of the crucial aspects of secondary studies is meta-epidemiology, also known as meta-research [7], which is the study of research itself, focusing on how studies are designed, conducted, and reported. Investigating these aspects is essential for understanding and improving the quality of Systematic Reviews and other secondary research methodologies. It was especially true during the COVID-19 pandemic when researchers conducting Systematic Reviews faced unprecedented challenges. Unlike before, they were required to rapidly and efficiently integrate data from unconventional sources such as data modelling studies, infection routes, and contact tracing. It marked a significant shift in the credibility and reliability of sources, necessitating new approaches to assess and synthesise this information.

This study aimed to evaluate the current practices, preferences, and challenges researchers face conducting secondary studies, focusing on Systematic Reviews during the COVID-19 pandemic. By assessing the impact of the pandemic on secondary research methodologies, the use of tools and guidelines, and the adoption of systematic approaches, this study sought to improve the quality and reliability of secondary research. Additionally, this research aimed to fill a knowledge gap by identifying key practices and challenges researchers encountered. It highlights areas for improvement, such as the adoption of keyword generation tools and protocol registration, offering valuable insights to enhance the quality of future secondary studies.

## 2. Materials and Methods

### 2.1. Study Design

A cross-sectional survey was employed in this research, as it facilitates the swift collection of data from a targeted cohort. By using a survey, the researchers aimed to gather a broad range of opinions, preferences, and levels of knowledge regarding the methodology of Systematic Reviews. The survey format is particularly suitable for this purpose because it can capture and reflect a broad spectrum of respondents’ experiences and practices.

This strategy is well suited for documenting and reflecting participants’ diverse experiences and behaviours. The findings were presented following the survey studies checklist [8].

### 2.2. Data Collection and Research Method

An anonymous survey (Supplementary File) was designed for respondents engaged in secondary studies—scientific researchers, clinicians, healthcare professionals, and medical students. Email addresses were obtained from the PubMed database. We applied criteria like the publication date (from 2020 to 2022), article type (Systematic Review), and keyword (“COVID-19”). After that, we were able to receive 2898 email addresses, of which 388 were unsuccessfully delivered. Finally, the target population was 2510. A total of 78 unique responses (only the corresponding author of each study) were obtained and subjected to statistical analysis. By distributing the survey via email to researchers identified through PubMed, this study reached individuals actively publishing in the field during a critical time (COVID-19 pandemic), allowing for timely and relevant insights.

A database of email addresses was compiled, and a research survey was sent out in three rounds. Most email addresses belonged to research directors and individuals holding professorial titles (corresponding authors).

Our survey focused on exploring the relationships between the frequency of conducted Systematic Reviews, their publication, selected methodologies and tools, and individual scientific interests and experiences related to the comprehensive process of conducting Systematic Reviews. These data were compared with those associated with the impact of the COVID-19 pandemic on the popularisation of secondary research.

The survey includes three sections: Section I, general information (comprising 4 single-choice questions and 2 open-ended questions); Section II, Systematic Review process (comprising 9 single-choice questions, 6 multiple-choice questions, 4 open-ended questions, and 3 Yes/No grid questions); and Section III, secondary studies during the COVID-19 pandemic (comprising 2 single-choice questions, 3 multiple-choice questions, and 1 open-ended question).

The survey was administered in English.

To avoid ambiguity, the term “Systematic Review” and related terminology (e.g., “Systematic Review software”) used throughout Section II of the survey was intended as an umbrella term referring to all structured forms of secondary research. This includes not only Systematic Reviews in the strict Cochrane sense, but also Scoping Reviews, Rapid Reviews, Umbrella Reviews, Evidence Maps, and Qualitative Systematic Reviews. In contrast, in Section III, which addressed experiences related specifically to COVID-19-related publications, the term “secondary studies” (including Systematic Reviews) was used to distinguish between various types of evidence synthesis.

### 2.3. Informed Consent Statement

Participants were informed about this study’s purpose, their rights, and the option to withdraw at any time. Participation in the survey was entirely voluntary and considered equivalent to providing consent for collecting, processing, and using their data for scientific purposes. All information provided by the participants was used in an anonymised form to ensure confidentiality.

### 2.4. Sample Characteristics

The minimal sample size of 78 participants was calculated based on the number of emails retrieved from PubMed (2898–388 were unsuccessfully delivered). The sample size was calculated using the Raosoft Calculator (www.raosoft.com (accessed on 9 January 2023)) with a 10% margin of error, 95% confidence level, 2510 population size, and 30–70% response distribution.

The adopted parameters were based on the assumption of a larger margin of error (10%) and a skewed response distribution (30–70%). The margin of error accounts for the loosely defined target population (corresponding authors of published studies) and the aim of obtaining results that indicate general trends within the target population. Regarding the response distribution, it was assumed that a significant portion of responses could be one-sided (e.g., in questions about guidelines or software used, most respondents are likely to provide similar answers).

### 2.5. Survey Administration

This study was conducted from 17 January 2023, to 31 March 2023. The survey was created and hosted using Google Forms. Subsequently, the questionnaire was distributed to researchers via publicly available emails, allowing it to reach researchers worldwide and send personalised invitations to this study.

### 2.6. Statistical Methods

The study results underwent statistical analysis using Pearson’s Chi^2^ test of independence. TIBCO Statistica 13.3 software was employed for analysis. A statistically significant value of the Pearson Chi^2^ coefficient was considered to be *p* < 0.05. Data analysis was conducted using Statistica 13.3 software and the MS Office (Excel) LTSC 2021 package. Additionally, the obtained results were subjected to descriptive statistics analysis. For the analysis of multiple-choice question data, each possible answer was converted into a binary variable (Yes/No) and then checked for correlations with other responses (Supplementary File). Responses with missing answers for a given variable were excluded from the corresponding analysis, and the effective sample size (n) was adjusted accordingly.

## 3. Results

### 3.1. Study Group Characteristics

The first section of the manuscript concerned a respondent’s general information (six questions). The results are presented in Table 1. A total of 78 individuals participated in this study.

### 3.2. Descriptive Survey Results (Section II and Section III)

The subsequent part of the survey presents data on conducting Systematic Reviews (Section II) and secondary studies (including Systematic Reviews) during the COVID-19 pandemic (Section III). The obtained results are presented in Table 2 (single-choice question results) and Table 3 (multiple-choice question results), as well as in four figures illustrating the distribution of responses to the Yes/No grid questions from Section II (7.2, 8.1, and 10) and one single-choice question from Section III (4).

### 3.3. Statistical Analysis

An in-depth statistical analysis of Pearson’s Chi^2^ dependencies between respondents’ answers was provided in Table 4 and Table 5 and in the Supplementary File (Table S1). The analysis covered two questions (questions 1 and 5) from Section I (general information), seventeen questions (excluding open-ended questions 5.1, 7.3, 8.2, and 10.1, and single-choice question 12) from Section II (Systematic Review process), as well as six questions from Section III (secondary studies during the COVID-19 pandemic).

## 4. Discussion

A Systematic Review and other secondary research constitute a demanding area of study that only seemingly requires less organisational and scientific involvement (compared to experimental research) in producing credible and high-quality results. The proper conduct of this process depends on the availability of scientific evidence and skills in acquiring and assessing their reliability, skilful data extraction, or summarising them in forms such as meta-analysis. It also depends on the tools facilitating the execution of this process or the ability to adapt specific secondary research guidelines for a particular type of study or research problem. The findings of this study reveal several important trends and correlations in secondary research practices, particularly in the context of the COVID-19 pandemic.

The results indicate that the most commonly used medical databases include PubMed, Cochrane Library, Scopus, and Embase. PubMed is one of the largest (37 million citations as of 2025) and most popular medical databases, providing access to bibliographic data, abstracts, and links to full-text publications [9]. This study also highlights the critical role of keyword-generation tools in literature searches. Researchers who frequently use PubMed are more likely to employ structured keyword tools like MeSH, which can improve search precision and comprehensiveness (*p* = 0.01486, df = 1, Chi^2^ = 5.932568). Creating a query and entering it into a search engine allows for precisely searching publications based on specific criteria [10,11]. However, the relatively low overall adoption of these tools (only 26.9% of respondents use them) suggests that many researchers rely on manual or less systematic approaches. This gap underscores the need for increased training and awareness of effective search strategies, as comprehensive literature retrieval is fundamental to high-quality secondary research.

Among the most popular types of secondary research indicated by respondents are Rapid, Systematic, and Scoping Reviews. A Rapid Review shares many characteristics of a typical Systematic Review, but it allows for obtaining final results in a shorter time by applying a series of simplifications in the analysis process (e.g., searching for publications in only one database or only in English, limited grey literature searching, or screening for most records conducted by one reviewer). This type of review is chosen when synthesised information based on scientific evidence is needed faster, more economically, and more timely [12]. A typical Systematic Review involves a comprehensive synthesis and analysis of data from included primary studies and the publication of evidence-based conclusions [13]. On the other hand, a Scoping Review allows greater flexibility as it considers the diversity of the literature and studies using various methodologies. It may include the grey literature, qualitative and quantitative research, and theoretical and narrative reviews [14]. According to Munn et al. [15], researchers may conduct a Scoping Review instead of a Systematic Review when the review’s objective is to identify knowledge gaps and the scope of the literature, explain concepts, or examine the research trajectory. A Systematic Review is recommended when we want to determine areas where evidence is lacking, which can help categorise future research in that field [16]. The significant association between researchers’ experience and their likelihood to conduct Scoping Reviews (*p* = 0.00738, df = 3, Chi^2^ = 12.00221) suggests that more seasoned researchers prefer this type of study for its flexibility and broad applicability. Scoping Reviews are beneficial in areas where evidence is sparse or diverse, allowing experienced researchers to map key concepts and identify gaps. This preference may also reflect the evolving complexity of research questions addressed by senior researchers, as their expertise enables them to handle more nuanced and exploratory topics.

**Figure 1:**
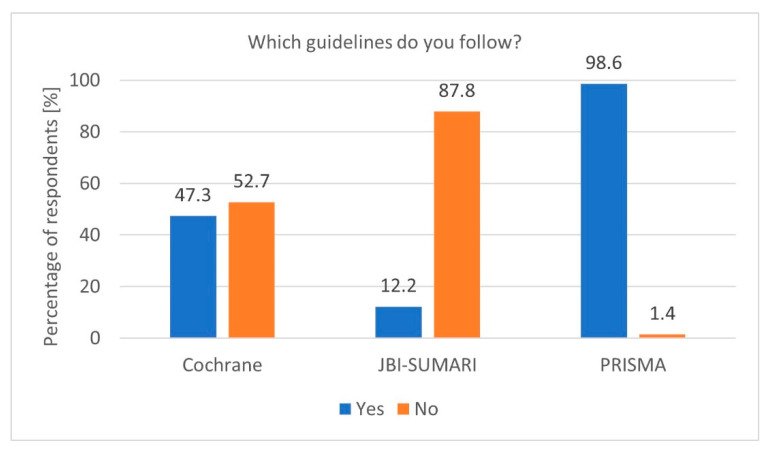
Most popular guidelines (n = 74).

To properly prepare a Systematic Review, it is essential to adhere to specific guidelines. Adherence to established guidelines, particularly PRISMA, is another notable finding. The high PRISMA [6] guideline usage rate among researchers conducting Systematic Reviews demonstrates a strong commitment to methodological standards (*p* = 0.02390, df = 1, Chi^2^ = 5.101622). The Cochrane Collaboration guidelines [5] and the Joanna Briggs Institute (JBI) [17] guidelines were less popular (Figure 1). Furthermore, researchers registering protocols are significantly more likely to follow PRISMA guidelines (*p* = 0.00320, df = 2, Chi^2^ = 11.48858). However, the occasional lack of adherence, indicated by the 5.1% of respondents who do not follow any guidelines, raises concerns about the quality and reproducibility of some reviews. Encouraging universal adherence to standardised guidelines, along with providing accessible training, could help address these issues.

The results of this study indicate that most of the researchers (our respondents) had the opportunity (in their careers) to conduct a Cochrane review, which is significant considering that reviews published in the Cochrane Database of Systematic Reviews are entirely based on a comprehensive and multi-faceted methodology published in the “Cochrane Handbook for Systematic Reviews of Interventions” [5] and thus considered the most reliable type of scientific evidence. Respondents conducting Cochrane Systematic Reviews were significantly more inclined to undertake Umbrella Reviews (*p* = 0.00395, df = 1, Chi^2^ = 8.308696), likely due to the comprehensive nature of Cochrane reviews, which often necessitate synthesis across multiple Systematic Reviews. Additionally, the association between experience and the likelihood of conducting Umbrella Reviews further emphasises the role of expertise in secondary research. Respondents experienced in Scoping Reviews and Evidence Maps were significantly more likely to conduct Umbrella Reviews (*p* = 0.03446, df = 1, Chi^2^ = 4.471640; *p* = 0.00224, df = 1, Chi^2^ = 9.343750).

Protocol registration is crucial, a step opted for by most respondents, which occurs in at least one of the dedicated repositories, such as PROSPERO (University of York) or in a selected scientific journal [18]. The most commonly used repositories include PROSPERO, OSF, and Cochrane. Interestingly, despite the general emphasis on methodological rigour in Systematic Reviews, not all researchers consistently register protocols. While those conducting Systematic Reviews are more likely to register their protocols (*p* = 0.00387, df = 2, Chi^2^ = 11.11158), this practice is less common among those conducting Scoping Reviews (*p* = 0.01958, df = 2, Chi^2^ = 7.866348). This discrepancy could stem from the exploratory nature of Scoping Reviews, where predefined methods are less rigid and flexibility is often necessary. However, the lack of protocol registration can compromise transparency and reproducibility. Encouraging broader adoption of protocol registration, even for Scoping Reviews, may enhance the credibility of these studies.

**Figure 2:**
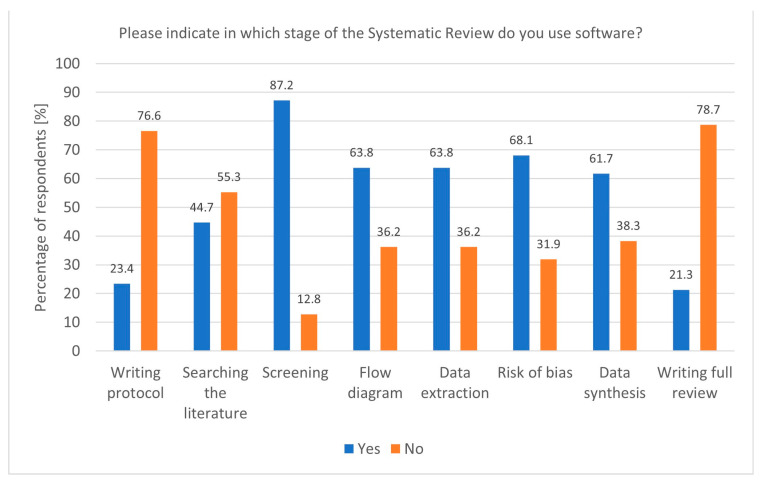
Stages of a Systematic Review and the utilisation of software (n = 47).

The widespread use of dedicated software for Systematic Reviews reflects the growing reliance on technological tools to enhance research efficiency. Tools like Rayyan and Covidence, particularly popular among researchers conducting Systematic and Rapid Reviews, streamline critical stages such as screening and data extraction. The association between Covidence use and Rapid Reviews (*p* = 0.00843, df = 1, Chi^2^ = 6.938953) underscores its value in time-sensitive research, where quick yet accurate synthesis is essential. Moreover, the fact that software is most commonly used during the screening stage (*p* = 0.02400, df = 1, Chi^2^ = 5.094851) aligns with the need to manage large volumes of the literature efficiently (Figure 2). In addition to screening, software was also popular during flow diagram creation, data extraction, risk of bias analysis, and data synthesis. Despite this, some researchers still do not use any software, highlighting a potential area for improvement in research practice. The Systematic Review (SR) Toolbox is also worth mentioning as a website that compiles links and information about tools used at each stage of a Systematic Review [19].

**Figure 3:**
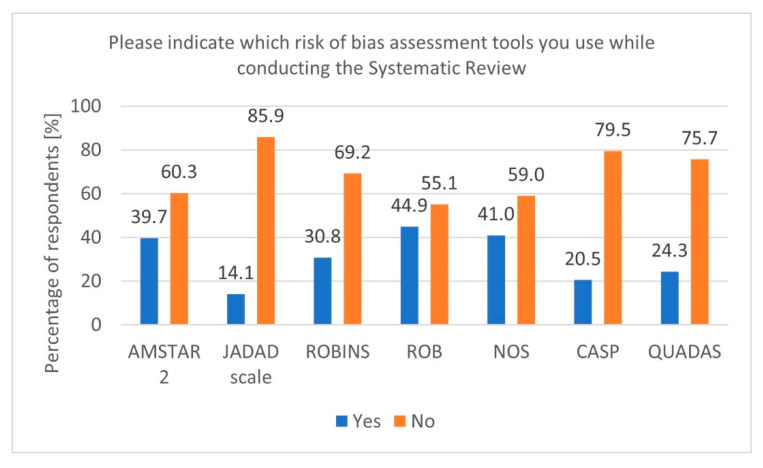
Risk of Bias tools (n = 78).

During secondary research, there is a risk of various biases. Therefore, using tools to verify and adequately assess this risk is essential [20]. In our survey, most respondents indicated using tools such as Cochrane RoB, Newcastle–Ottawa Scale (NOS), and A MeaSurement Tool to Assess Systematic Reviews 2 (AMSTAR 2) (Figure 3). Additionally, it is worth mentioning that for experimental studies involving animals, a dedicated tool called SYRCLE was developed based on the original Cochrane RoB tool for RCT studies [21].

**Figure 4:**
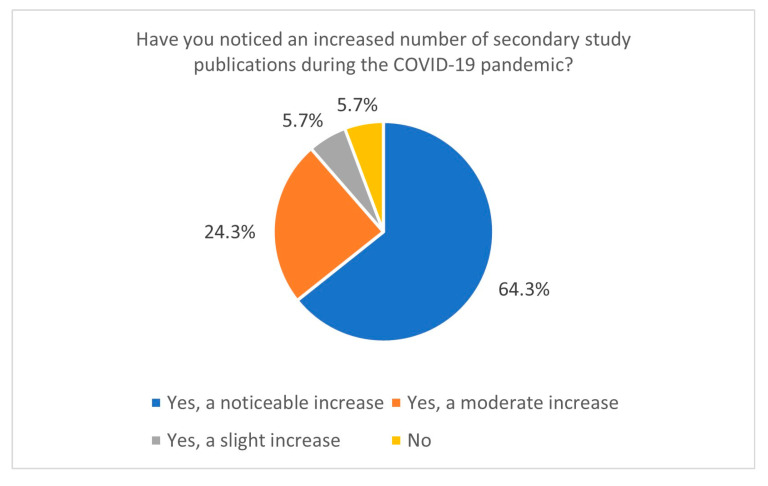
The number of secondary study publications increased during the COVID-19 pandemic (n = 70).

This study also sheds light on research practices during the COVID-19 pandemic. The increased publication of secondary studies during the pandemic, particularly between January and June 2021, indicates a heightened demand for synthesised evidence in response to the global health crisis. Most respondents reported conducting Systematic Reviews during this period, and 64.3% observed a significant increase in the publication of secondary studies related to COVID-19 (Figure 4). It correlates with data directly from the PubMed database—using the keyword “COVID-19” yields 141,684 records, giving us around 388 papers per day in 2021 (PubMed data, 15 January 2025). This trend likely resulted from the urgent need for actionable insights to guide clinical and public health decisions. However, the accelerated publication process during the pandemic, including the rise of preprints [22,23,24], has raised concerns about the quality and reliability of some studies. Preprints on COVID-19 ultimately constituted a significant portion of the pool of withdrawn articles in 2020 [25,26]. Nevertheless, in 2020, over 30,000 preprints on COVID-19 topics were published, accounting for approximately 30% of all published articles on COVID-19 during that time. Articles related to COVID-19 were also more frequently cited and shared [23].

The preference for high-quality evidence, such as randomised controlled trials (RCTs), in Systematic Reviews, is consistent with the principles of evidence-based medicine. The significant correlation between Embase use and the incorporation of RCTs during COVID-19 (*p* = 0.00360, df = 1, Chi^2^ = 8.476092) suggests that researchers prioritising rigorous evidence tend to use specialised databases with comprehensive indexing of clinical trials. Additionally, respondents conducting Systematic Reviews were significantly more likely to incorporate RCTs in this mentioned period (*p* = 0.01873, df = 1, Chi^2^ = 5.526316). However, the low usage of non-randomised trials by researchers conducting Cochrane reviews (*p* = 0.02601, df = 1, Chi^2^ = 4.955580) reflects the stringent inclusion criteria and high methodological standards upheld by Cochrane. An analysis also revealed a significant association between Web of Science use and non-randomised trials (*p* = 0.04826, df = 1, Chi^2^ = 3.900929). This could indicate that Web of Science users have a broader approach to evidence synthesis, incorporating observational studies alongside experimental research. Similarly, a strong correlation was observed between Scopus usage and case–control studies (*p* = 0.00841, df = 1, Chi^2^ = 6.944445). This finding may reflect Scopus’s extensive indexing of epidemiological and observational studies, which are often employed in case–control research. Likewise, PubMed users preferred cross-sectional studies (*p* = 0.00265, df = 1, Chi^2^ = 9.032258), which aligns with the database’s well-established role in providing access to a vast range of the biomedical literature, including descriptive and prevalence-based research.

## 5. Limitations

Despite the valuable insights provided by this study, several limitations should be noted. Some statistical results may be affected by imbalanced group sizes, especially in cases where only a small number of participants selected a given response option. Such asymmetry can limit the validity of statistical significance tests and increase the likelihood of random variation influencing the outcomes. Therefore, specific associations identified in this study should be interpreted with caution. Future research should aim to include larger and more evenly distributed samples to enhance the robustness and generalisability of the findings. Furthermore, while Pearson’s Chi^2^ test explored associations between categorical variables, we acknowledge that more advanced techniques—such as Latent Class Analysis (LCA)—could provide deeper insight into patterns within multiple-choice responses. However, due to the limited sample size and this study’s exploratory nature, such modelling approaches were not applied.

Additionally, the respondents were predominantly experienced researchers, which may have introduced a bias towards more established practices. The geographic diversity of respondents could also imply variations in access to resources and training, potentially influencing the reported practices. Furthermore, open-ended responses were sometimes challenging to interpret accurately, which may have affected the analysis.

## 6. Conclusions

In conclusion, this study highlights key practices, preferences, and challenges in secondary research. While adherence to guidelines and using advanced tools are prevalent among researchers, gaps remain in protocol registration, keyword generation, and software adoption. Addressing these gaps through targeted training and increased awareness could enhance the quality of secondary research. The findings also underscore the importance of maintaining methodological rigour, especially during global crises like the COVID-19 pandemic, where the demand for reliable evidence is paramount.

## Figures and Tables

**Table 1:** Study population—general information.

Question	Answers	Result [%]
What is your age? (In years) (n = 78)	20–29	11.5
	30–39	33.3
	40–49	23.1
	50–59	16.7
	60–69	11.5
	70–79	2.6
	>80	1.3
What is your gender? (n = 78)	Woman	38.4
	Man	59.0
	Non-binary	1.3
	I prefer not to answer	1.3
In which country do you work? (n = 78)	UK	10.3
	India	10.3
	US	9.0
	Iran	7.7
	Australia	6.4
	Canada	6.4
	Indonesia	5.1
	Brazil	3.8
	Germany	3.8
	Spain	3.8
	Denmark	2.6
	Ethiopia	2.6
	Italy	2.6
	Philippines	2.6
	Sweden	2.6
	Others	20.4
What is your current occupational position/title? (n = 78)	Professor	25.6
	Associate Professor	17.9
	Researcher in academia/industry/non-academic organisations	15.4
	Assistant Professor	11.5
	Postdoc	10.3
	Student	6.4
	Clinician	5.1
	Others	7.8
How experienced are you in the secondary studies field (in years)? (n = 78)	Less than a year	3.8
	1–5 years	20.5
	5–10 years	30.8
	>10 years	44.9

**Table 2:** Summary of responses to single-choice questions.

Section	Question Number	Question	Answer	Result (%)
2	2 (n = 78)	Do you use literature management software?	Always	61.1
			Sometimes	29.1
			Never	9.8
2	4 (n = 78)	Do you register the protocol for secondary studies?	Yes, always	41.0
			Sometimes	48.7
			No, never	10.3
2	5 (n = 78)	Have you conducted a Cochrane review yet?	Yes	16.7
			No	83.3
2	6 (n = 78)	Do you use tools for creating keyword series?	Yes	26.9
			No	73.1
2	7 (n = 78)	Do you use software for a Systematic Review?	Yes	60.3
			No	39.7
2	8 (n = 78)	Do you follow any guidelines when conducting a Systematic Review?	Yes	94.9
			No	5.1
2	9 (n = 78)	Do you perform a Systematic Review of other (published) Systematic Reviews?	Yes	41.0
			No	59.0
2	11 (n = 78)	Do you perform the GRADE assessment for your Systematic Review?	Yes	42.3
			No	35.9
			Sometimes	21.8
2	12 (n = 78)	Are you an author of any published secondary study related to COVID-19?	Yes	89.7
			No	10.3
3	4 (n = 70)	Did you publish your work as a preprint?	Yes	32.9
			No	67.1
3	5 (n = 68)	What was the time (in months) between submission and publishing the COVID-19-related paper (papers)? (Please describe every study) (open question)	1–3	32.3
			4–6	32.3
			7–9	16.2
			10–12	14.7
			>12	4.5

**Table 3:** Summary of responses to multiple-choice questions.

Section	Question Number	Question	Answer	Result (%)
2	1 (n = 78)	Which medical databases do you use during the development of Systematic Reviews?	PubMed	94.9
			Cochrane Library	69.2
			Scopus	69.2
			Embase	65.4
			Web of Science	62.8
			PubMed Central	48.7
			Science Direct	39.7
			PsychInfo	23.1
			Others	16.7
2	2.1 (n = 78)	Choose the software which you use	EndNote	67.1
			Mendeley	41.4
			Zotero	18.6
			Covidence	11.4
			Others	20.0
2	3 (n = 78)	Which types of secondary studies did you prepare in the past?	Systematic Review	97.4
			Scoping Review	57.7
			Rapid Review	37.2
			Qualitative Systematic Review	29.5
			Umbrella Review	23.1
			Evidence Map	7.7
			Meta-analysis	3.8
			Narrative review	1.3
2	4.1 (n = 70)	On which websites do you upload the Systematic Review protocol?	PROSPERO	71.4
			Cochrane.org	20.0
			Osf.io	20.0
			Dataryad.org	5.7
			Others	10.0
2	6.1 (n = 21)	Which tools do you use for creating keyword series?	MeSH	100.0
			Emtree	19.0
			CD-10	9.5
			MedDRA	4.8
			Cumulated Index of Nursing and Allied Health Literature (CINAHL)	4.8
2	7.1 (n = 48)	Which software for a Systematic Review do you use?	Rayyan	39.6
			Covidence	37.5
			RevMan	16.7
			SysRev	12.5
			DistillerSR	10.4
			SR-Accelerator	10.4
			Excel (Meta)/Google Sheets	8.3
			RobotReviewer	8.3
			EPPI-Reviewer Web	6.2
			JBI SUMARI	4.2
			PICOPortal	4.2
			Others	12.5
3	1 (n = 70)	At which stage of the COVID-19 pandemic did you prepare a secondary study (studies)?	January 2020–June 2020	34.3
			July 2020–December 2020	38.6
			January 2021–June 2021	41.4
			July 2021–December 2021	35.7
			January 2022–June 2022	22.9
			July 2022–December 2022	17.1
3	3 (n = 70)	Which type of evidence did you prefer to incorporate into your secondary study (studies) conducted during the COVID-19 pandemic (2020–2022)?	Randomised trials [Randomised controlled trials (RCTs)]	72.9
			Cohort studies	60.0
			Cross-sectional study	50.0
			Case–control study	42.9
			Systematic Reviews	31.4
			Non-randomised trials	27.1
			Case series or case studies	18.6
			Single-arm trials	10
			Modelling studies	7.1
			Other	7.1
3	6 (n = 70)	Which publisher published your paper related to the COVID-19 subject?	Elsevier	40.6
			Biomed Central (BMC)	30.4
			Springer	24.6
			Wiley	14.5
			MDPI	13.0
			BMJ (BMJ Open, BMJ Journals)	8.7
			Nature	8.7
			Taylor & Francis	5.8
			Others	20.3

**Table 4:** Statistical analysis of selected results (Section II).

Question	Question VERSUS Question (Section Number/Question Number)	Correlation	Possible Interpretation
Which types of secondary studies did you prepare in the past?	Scoping Review (II/3) vs. Experience (I/5) *p* = 0.00738 df = 3 Chi^2^ = 12.00221	Conducted Scoping Reviews vs. secondary studies experience	With experience, the frequency of conducting Scoping Reviews increases
Do you register the protocol for secondary studies?	Yes, always/Sometimes (II/4) vs. Systematic Review (II/3) *p* = 0.00387 df = 2 Chi^2^ = 11.11158	Registering protocol vs. conducting Systematic Reviews	Respondents conducting Systematic Reviews often register their study protocols
	Yes, always/Sometimes (II/4) vs. Scoping Review (II/3) *p* = 0.01958 df = 2 Chi^2^ = 7.866348	Registering protocol vs. conducting Systematic Reviews	Respondents conducting Scoping Reviews rarely register their study protocols
Do you use tools for creating keyword series?	Yes (II/6) vs. PubMed Central (II/1) *p* = 0.01486 df = 1 Chi^2^ = 5.932568	Using keyword generation tools vs. using PubMed Central	Respondents using PubMed Central often utilise keyword generation tools
Do you use software for Systematic Review?	Yes (II/7) vs. Systematic Review (II/3) *p* = 0.02964 df = 1 Chi^2^ = 4.730322	Using dedicated software vs conducting Systematic Reviews	Respondents conducting Systematic Reviews often use dedicated software
Which software for Systematic Review do you use?	Covidence (II/7.1) vs. Rapid Review (II/1) *p* = 0.00843 df = 1 Chi^2^ = 6.938953	Using Covidence vs. conducting Rapid Reviews	Respondents conducting Rapid Reviews often use the Covidence software (https://www.covidence.org/)
	Covidence (II/7.1) vs. Yes (II/5) *p* = 0.01906 df = 1 Chi^2^ = 5.495800	Using Covidence vs. conducting Cochrane Systematic Reviews	Respondents conducting Cochrane Systematic Reviews often use the Covidence software
Please indicate in which stage of the Systematic Review do you use the software?	Screening (II/7.2) vs. Rapid Review (II/3) *p* = 0.02400 df = 1 Chi^2^ = 5.094851	Using software during screening vs. conducting Rapid Reviews	Respondents conducting Rapid Reviews often use software at the screening stage
Do you follow any guidelines when conducting a Systematic Review?	Yes (II/8) vs. Systematic Review (II/3) *p* = 0.02390 df = 1 Chi^2^ = 5.101622	Following guidelines vs. conducting Systematic Reviews	Respondents conducting Systematic Reviews often use guidelines
Which guidelines do you follow?	PRISMA (II/8.1) vs. Systematic Review (II/3) *p* = 0.00000 df = 1 Chi^2^ = 36.49315	Following PRISMA guidelines vs. conducting Systematic Reviews	Respondents conducting Systematic Reviews often use PRISMA guidelines
	PRISMA (II/8.1) vs. Yes, always/Sometimes (II/4) *p* = 0.00320 df = 2 Chi^2^ = 11.48858	Following PRISMA guidelines vs. registering study protocols	Respondents registering study protocols often use PRISMA guidelines
Do you perform Systematic Reviews of other (published) Systematic Reviews?	Yes (II/9) vs. Scoping Review (II/3) *p* = 0.03446 df = 1 Chi^2^ = 4.471640	Conducting Umbrella Reviews vs. conducting Scoping Reviews	Respondents experienced in Scoping Reviews often conduct Umbrella Reviews
	Yes (II/9) vs. Evidence Map (II/3) *p* = 0.00224 df = 1 Chi^2^ = 9.343750	Conducting Umbrella Reviews vs. conducting Evidence Map	Respondents experienced in Evidence Maps often conduct Umbrella Reviews
	Yes (II/9) vs. Yes (II/5) *p* = 0.00395 df = 1 Chi^2^ = 8.308696	Conducting Umbrella Reviews vs. conducting Cochrane Systematic Reviews	Respondents conducting Cochrane Systematic Reviews often conduct Umbrella Reviews

**Table 5:** Statistical analysis of selected results (Section III).

Question	Question VERSUS Question (Section Number/Question Number)	Correlation	Possible Interpretation
Which type of evidence did you prefer to incorporate into your secondary study (studies) conducted during the COVID-19 pandemic (2020–2022)?	Randomised trials (III/3) vs. Embase (II/1) *p* = 0.00360 df = 1 Chi^2^ = 8.476092	Preference of randomised trials vs. using Embase	Respondents using the Embase database often use randomised trials
	Non-randomised trials (III/3) vs. Web of Science (II/1) *p* = 0.04826 df = 1 Chi^2^ = 3.900929	Preference of non-randomised trials vs. using Web of Science	Respondents using the Web of Science database often use randomised trials
	Case–control (III/3) vs. Scopus (II/1) *p* = 0.00841 df = 1 Chi^2^ = 6.944445	Preference of case–control studies vs. using Scopus	Respondents using the Scopus database often use case–control studies
	Cross-sectional (III/3) vs. PubMed (II/1) *p* = 0.00265 df = 1 Chi^2^ = 9.032258	Preference of cross-sectional studies vs. using PubMed	Respondents using the PubMed database often use cross-sectional studies
	Randomised trials (III/3) vs. Systematic Review (II/3) *p* = 0.01873 df = 1 Chi^2^ = 5.526316	Preference of randomised trials vs. conducting Systematic Reviews	Respondents conducting Systematic Reviews often use randomised trials
	Non-randomised trials (III/3) vs. No (II/5) *p* = 0.02601 df = 1 Chi^2^ = 4.955580	Preference of non-randomised trials vs. conducting Cochrane Systematic Reviews	Respondents conducting Cochrane Systematic Reviews rarely use non-randomised trials

## Data Availability

The original contributions presented in this study are included in the article/Supplementary Materials. Further inquiries can be directed to the corresponding author(s).

## Supplementary Materials

The following supporting information can be downloaded at https://www.mdpi.com/article/10.3390/healthcare13080927/s1, Supplementary Information, including a survey questionnaire, additional statistical analysis results, and raw data.
