# Supplementary material for: The Perspective on Secondary Research Practices: A Cross-Sectional Analysis
Source: Healthcare (Basel). 2025 Apr 17;13(8):927. doi: 10.3390/healthcare13080927 (PMC12027147; doi:10.3390/healthcare13080927)
Supplement: Supplementary file 1 [file healthcare-13-00927-s001.zip › Supplementary information.pdf]

Table S1. Statistical Analysis of Survey Questions.

Question	Question VERSUS question (section number/question number)	Correlation	Possible interpretation
**Which types of secondary studies did you prepare in the past?**	Systematic Review (II/3) vs EndNote (II/2.1)<br>**p=0.04025**<br>df=1<br>Chi^2=4.207161	Conducted Systematic Reviews vs using EndNote	Respondents conducting a Systematic Review often use EndNote
	Systematic Review (II/3) vs Embase (II/1)<br>**p=0.01520**<br>df=1<br>Chi^2=5.893333	Conducted Systematic Reviews vs using Embase	Respondents conducting a Systematic Review often use the Embase database
	Scoping Review (II/3) vs Age (I/1)<br>**p=0.04047**<br>df=6<br>Chi^2=13.16633	Conducted Scoping Reviews vs age	The frequency of conducting Scoping Reviews increases with age
	Scoping Review vs EndNote (II/2.1)<br>**p=0.00041**<br>df1<br>Chi^2=12.47610	Conducted Scoping Reviews vs using EndNote	Respondents conducting Scoping Reviews often use EndNote
	Rapid Review (II/3) vs EndNote (II/2.1)<br>**p=0.01673**<br>df=1<br>Chi^2=5.724012	Conducted Rapid Reviews vs using EndNote	Respondents conducting Rapid Reviews often use EndNote
	Qualitative SR (II/3) vs Embase (II/1)<br>**p=0.00186**<br>df=1<br>Chi^2=9.682244	Conducted Qualitative Systematic Reviews vs using Embase	Respondents conducting Qualitative Systematic Reviews often use the Embase database
	Qualitative SR (II/3) vs EndNote (II/2.1)<br>**p=0.00261**<br>df=1<br>Chi^2=9.064542	Conducted Qualitative Systematic Reviews vs using EndNote	Respondents conducting Qualitative Systematic Reviews often use EndNote
	Umbrella Review (II/3) vs EndNote (II/2.1)<br>**p=0.02267**<br>df=1<br>Chi^2=5.193948	Conducted Umbrella Reviews vs using EndNote	Respondents conducting Umbrella Reviews often use EndNote
**On which websites do you upload the Systematic Review protocol?**	Cochrane.org (II/4.1) vs Systematic Review (II/3)<br>**p=0.04396**<br>df=1<br>Chi^2=4.057971	Using Cochrane.org repository vs conducting Systematic Reviews	Respondents conducting Systematic Reviews rarely register their study protocols on the Cochrane.org website
**Do you use software for Systematic Review?**	Yes (II/7) vs Scoping Review (II/3)<br>**p=0.02216**<br>df=1<br>Chi^2=5.233094	Using dedicated software vs conducting Systematic Reviews	Respondents conducting Scoping Reviews often use dedicated software
**Which software for Systematic Review do you use?**	Rayyan (II/7.1) vs Age (I/1)<br>**p=0.03457**<br>df=5<br>Chi^2=12.01584	Using Rayyan software vs age	Younger respondents more frequently use the Rayyan software
	SysRev (II/7.1) vs Rapid Review (II/1)<br>**p=0.04177**<br>df=1<br>Chi^2=4.144621	Using SysRev software vs conducting Rapid Reviews	Respondents conducting Rapid Reviews rarely use the SysRev software
	EPPI-Reviewer Web (II/7.1) vs Rapid Review (II/1)<br>**p=0.03753**<br>df=1	Using EPPI-Reviewer Web software vs conducting Rapid Reviews	Respondents conducting Rapid Reviews rarely use the EPPI-Reviewer Web software

	Chi^2=4.326137		
	Covidence (II/7.1) vs Qualitative Systematic Review (II/1) **p=0.00616** df=1 Chi^2=7.503193	Using Covidence software vs conducting Qualitative Systematic Reviews	Respondents conducting Qualitative Systematic Reviews rarely use the Covidence software
	SR-Accelerator (II/7.1) vs Umbrella Review (II/1) **p=0.00214** df=1 Chi^2=9.429196	Using SR-Accelerator software vs conducting Umbrella Reviews	Respondents conducting Umbrella Reviews rarely use the SR-Accelerator software
	Robot Reviewer (II/7.1) vs Umbrella Review (II/1) **p=0.03872** df=1 Chi^2=4.273319	Using Robot Reviewer software vs conducting Umbrella Reviews	Respondents conducting Umbrella Reviews rarely use the Robot Reviewer software
**Do you follow any guidelines when conducting a Systematic Review?**	Yes (II/8) vs Yes (II/4) **p=0.01337** df=2 Chi^2=8.630068	Following guidelines vs registering study protocol	Respondents registering study protocols often use guidelines
**Which guidelines do you follow?**	Cochrane (II/8.1) vs Qualitative SR (II/3) **p=0.03567** df=1 Chi^2=4.413030	Following Cochrane guidelines vs conducting Qualitative Systematic Reviews	Respondents conducting Qualitative Systematic Reviews often use Cochrane guidelines
**Please indicate which risk of bias assessment tools you use while conducting the Systematic Review**	AMSTAR (II/10) vs Scoping Review (II/3) **p=0.00418** df=1 Chi^2=8.202483	Using AMSTAR tool vs conducting Scoping Reviews	Respondents conducting Scoping Reviews often use the AMSTAR scale
	AMSTAR (II/10) vs Rapid Review (II/3) **p=0.03218** df=1 Chi^2=4.588723	Using AMSTAR tool vs conducting Rapid Reviews	Respondents conducting Rapid Reviews rarely use the AMSTAR scale
	AMSTAR (II/10) vs Umbrella Review (II/3) **p=0.00000** df=1 Chi^2=29.23706	Using AMSTAR tool vs conducting Umbrella Reviews	Respondents conducting Umbrella Reviews often use the AMSTAR scale
	AMSTAR (II/10) vs Evidence map (II/3) **p=0.00169** df=1 Chi^2=9.854838	Using AMSTAR tool vs conducting Evidence Maps	Respondents conducting Evidence Maps often use the AMSTAR scale
	AMSTAR (II/10) vs Yes (II/5) **p=0.00269** df=1 Chi^2=9.004530	Using AMSTAR tool vs conducting Cochrane Systematic Reviews	Respondents conducting Cochrane Systematic Reviews often use the AMSTAR scale
	NOS (II/10) vs Umbrella Review (II/3) **p=0.04824** df=1 Chi^2=3.901766	Using NOS tool vs conducting Umbrella Reviews	Respondents conducting Umbrella Reviews often use the NOS scale
	NOS (II/10) vs Yes (II/5) **p=0.02352** df=1 Chi^2=5.129348	Using NOS tool vs conducting Cochrane Systematic Reviews	Respondents conducting Cochrane Systematic Reviews often use the NOS scale
	CASP (II/10) vs Scoping Review (II/3) **p=0.03241** df=1 Chi^2=4.576686	Using CASP tool vs conducting Scoping Reviews	Respondents conducting Scoping Reviews rarely use the CASP scale

Link to the survey in google forms: https://bit.ly/3QKb2s6

SURVEY

COVID-19 pandemic impact on the Systematic Reviews popularisation.

Dear Researcher,

This short survey explores individual approaches and methods used to conduct a Systematic Review. The survey also contains questions about preparing publications during the COVID-19 pandemic. This survey also aims to measure how the COVID-19 pandemic influenced the researchers' preferences in perceiving and conducting Systematic Reviews.

This survey contains 24 questions, preferably taking 10 minutes to complete.

Please complete the survey before the March 31 2023, deadline.

Our survey is confidential, and no personal data will be publicly published. All data will be analysed and worked on collectively and, in this form, will be reported. All data will be curated by the Department of Pharmacoeconomics and Social Pharmacy at Poznan University of Medical Sciences (Poland).

The information you provide will be used solely in anonymised form. We use a publicly available email address to send personalised invitations to the study. Participation in the survey is voluntary.

The persons responsible for the study are Piotr Ratajczak (p_ratajczak@ump.edu.pl), PhD and Katarzyna Oziewicz (kasia.oziewicz@gmail.com).

This survey is a part of a Master's thesis written by Katarzyna Oziewicz under the supervision of Mr Piotr Ratajczak, PhD.

By participating in the survey, you agree to the electronic storage and use of anonymised data for scientific research.

GENERAL INFORMATION (SECTION I)

1. **What is your age? (open question)**
2. **What is your gender?**
    a. Woman
    b. Man
    c. Non-binary
    d. I prefer not to answer
    e. Other…

3. **In which country do you work? (open question)**

4. **What is your current occupational position/title?**
    a. Student
    b. Postdoc
    c. Associate Professor
    d. Assistant Professor
    e. Professor
    f. Researcher in academia/industry/non-academic organisations
    g. Clinician
    h. Other…

5. **How much experience are you in the secondary studies field (in years)?**
    a. less than a year
    b. 1-5 years
    c. 5-10 years
    d. >10 years

6. **What is your research area of interest? (open question)**

<u>SYSTEMATIC REVIEW PROCESS (SECTION II)</u>

1. **Which medical databases do you use during the development of systematic reviews (You may choose multiple answers)**
    a. Cochrane Library
    b. EMBASE
    c. PubMed
    d. PubMed Central
    e. Science Direct
    f. Scopus
    g. Web of Science
    h. PsychInfo
    i. Other…

2. **Do you use literature management software?**
    a. Always
    b. Sometimes
    c. Never

    2.1 Sub question to question 2 If 2-a/b (Always/Sometimes)
    If 2- a/b, Choose the software which you use:
    a. BibDesk
    b. Citavi
    c. EndNote
    d. JabRef
    e. Mendeley
    f. Papers
    g. Paperpile
    h. Qiqqa
    i. ReadCube
    j. RefWorks
    k. Sciwheel
    l. Zotero
    m. Other…

3. **Which types of secondary studies did you prepare in the past (You may choose multiple answers):**
    a. Systematic Review
    b. Scoping Review
    c. Rapid Review
    d. Qualitative Systematic Review
    e. Umbrella Review
    f. Evidence map
    g. Other…

4. **Do you register the protocol for secondary studies?**
    a. Yes, always
    b. Sometimes
    c. No, never

4.1. Sub question If 4-a/b(Yes, always; Sometimes)
On which websites do you upload the Systematic Review protocol? (You may choose multiple answers)

a. cochrane .org
b. crd. york .ac .uk/prospero/
c. datadryad .org
d. dataverse.harvard .edu
e. data.mendeley .com
f. figshare .com
g. osf .io
h. zenodo .org
i. vivli .org
j. Other…

5. **Have you conducted a Cochrane review yet?**
   a. Yes
   b. No

Sub question to 5 - Yes
5.a How many reviews have you published at Cochrane Library? (Open question)

6. **Do you use tools for creating keyword series?**
   a. Yes
   b. No

Sub question to 6-Yes
6.1 Which tools do you use for creating keyword series? (You may choose multiple answers)

a. AIHW
b. Australian Doctor Command Language (DOGLE)
c. Australian Medicines Terminology
d. MedDRA
e. CD-10
f. Cumulated Index of Nursing and Allied Health Literature (CINAHL)
g. Emtree
h. LOINC
i. MeSH
j. Ontology for biomedical investigations (OBI)
k. OpenGALEN
l. PLANETREE
m. RxNorm
n. SNOMED CT
o. Victorian Admitted Episodes Dataset (VAED)
p. Other…

7. Do you use software for Systematic Review?
   a. Yes
   b. No

<u>Sub questions if 7-Yes</u>
7.1 Which software for Systematic Review do you use? (You may choose multiple answers)

a. Cadima
b. Colandr
c. Covidence
d. Data Abstraction Assistant
e. DistillerSR
f. EPPI-Reviewer Web
g. Giotto Compliance

h. JBI SUMARI
i. LitStream
j. Nested Knowledge
k. PICOPortal
l. RobotAnalyst
m. RobotReviewer
n. Rayyan
o. SR-Accelerator
p. SRDB. PRO
q. SRDR /SRDR+
r. SyRF
s. SysRev
t. SWIFT-Activate Screener
u. Other…

7.2 Please indicate in which stage of the Systematic Review do you use the software?

Software	YES	NO
Writing protocol		
Searching literature		
Screening		
Flow diagram		
Data extraction		
Risk of bias		
Data synthesis		
Writing full review		

7.3 If you use the software in other stages/actions, please name it (open question)

8. Do you follow any guidelines when conducting a Systematic Review?

a. Yes
b. No

Sub questions if 8-Yes
8.1 Which guidelines do you follow?

GUIDANCE	YES	NO
Cochrane guidelines?		
JBI – SUMARI guidelines?		
PRISMA guidelines?		

8.2 If you use other guidelines, please name it (open question)

9. **Do you perform Systematic Reviews of other (published) Systematic Reviews?**
   a. Yes
   b. No

10. **Please indicate which risk of bias assessment tools you use while conducting the Systematic Review**

Assessment tool	YES	NO
AMSTAR 2 (Assessing the Methodological Quality of Systematic Review)		
JADAD scale		
ROBIS (Risk of Bias in Systematic Reviews)		
ROB (Risk of Bias)		
NOS (The Newcastle-Ottawa Scale for cohort and case-control studies)		
CASP (Critical Appraisal Skills Programme) Systematic Review checklist		
QUADAS-2 (A quality assessment tool for diagnostic accuracy studies)		

10.1 If you use other risk of bias assessment tools, please name them (open question)

11. **Do you perform the GRADE assessment (The Grading of Recommendations Assessment, Development and Evaluation) for your Systematic Reviews?**
   a. Yes
   b. No
   c. Sometimes

12. **Are you an author of any published secondary study related to COVID-19?**
   a. Yes
   b. No

SECONDARY STUDIES DURING THE COVID-19 PANDEMIC (SECTION III)

1. **At which stage of the COVID-19 pandemic did you prepare a secondary study (studies) (e.g. Systematic Review)? (You may choose multiple answers)**
   a. January 2020 – June 2020
   b. July 2020 – December 2020
   c. January 2021 – June 2021
   d. July 2021 – December 2021
   e. January 2022 – June 2022
   f. July 2022 – December 2022

2. **Have you noticed an increased number of secondary studies publications (e.g. systematic reviews, rapid reviews, scoping reviews) during the COVID-19 pandemic?**
   a. Yes, a noticeable increase
   b. Yes, a moderate increase
   c. Yes, a slight increase
   d. No
   e. Other…

3. **Which type of evidence did you prefer to incorporate into your secondary study (studies) conducted during the COVID-19 pandemic (2020 - 2022)? (You may choose multiple answers)**
   a. Randomised trials [Randomised controlled trials (RCT)]
   b. Non-randomised trials

c. Cohort studies
d. Case-control study
e. Cross-sectional study
f. Single-arm trials
g. Case series or case studies
h. Systematic Reviews
i. Modelling studies
j. Other…

4. **Did you publish your work as a preprint (pre-version of a scientific paper which has not been reviewed and published in a scientific journal) ?**
    a. Yes
    b. No

5. **What was the time (in months) between submission and publishing the COVID-19-related paper (papers)? (please describe every study) (open question)**

6. **Which publisher published your paper related to the COVID-19 subject? (You may choose multiple answers)**
    a. Biomed Central (BMC)
    b. Elsevier
    c. MDPI
    d. Nature
    e. Royal Society Publishing
    f. RSC
    g. Springer
    h. Taylor&Francis
    i. Wiley
    j. Other…

Insights/ opinions on this survey

If you have any suggestions on what could have been improved in this survey or want to leave an opinion, please share it in the box below
